# A longitudinal evaluation on 3-year change of anxiety and depression, and their risk factors among parents of childhood and adolescence patients with resectable osteosarcoma: A cohort study

**DOI:** 10.1097/MD.0000000000030981

**Published:** 2022-10-21

**Authors:** Jie Meng, Jing Wu, Xinying Zhang, Libo Guo, Honghe Li

**Affiliations:** a Department of Orthopedic Surgery, Harbin Medical University Cancer Hospital, Harbin, China.

**Keywords:** anxiety, depression, longitudinal change, parents of patients with osteosarcoma, risk factors

## Abstract

Psychological disorders often occur among parents of children with cancer. The current study aimed to explore the longitudinal change of anxiety and depression and their related factors among parents of childhood and adolescence patients with osteosarcoma. A total of 56 childhood and adolescence patients with osteosarcoma who underwent tumor resection and corresponding 104 parents were enrolled. Hospital Anxiety and Depression Scale-Anxiety (HADS-A) and HADS-Depression (HADS-D) of parents were evaluated at baseline (the day of patients’ hospital discharge), 0.5 year, 1 year, 2 years, and 3 years. From baseline to the 3^rd^ year, HADS-A (from 8.3 ± 3.1 to 9.4 ± 3.1. *P* < .001), HADS-D score (from 7.7 ± 3.2 to 8.8 ± 2.9, *P* = .001), anxiety rate (from 45.2% to 60.6%, *P* = .038), depression rate (from 38.5% to 57.7%, *P* = .002) were elevated; meanwhile, anxiety severity (*P* = .001) and depression severity (*P* = .001) were also increased. Furthermore, multivariate logistic regression analysis presented that the role of mother, divorced/widowed marital status, declined family annual income, elevated Enneking stage, and amputation were independently correlated with elevated risk of parents’ baseline anxiety or depression (all *P* < .05). Additionally, declined family annual income, elevated Enneking stage, and amputation were independently correlated with increased risk of parents’ 3-year anxiety or depression (all *P* < .05). Anxiety and depression deteriorate with time in parents of childhood and adolescence patients with osteosarcoma, which are affected by parental role, marital status, family annual income, surgery type, and Enneking stage.

## 1. Introduction

Osteosarcoma is an aggressive primary malignant tumor of bone, which originates from primary osteoblast mesenchymal cells and then develops into malignant osteoid.^[1]^ Although the overall incidence of osteosarcoma is low, osteosarcoma is viewed as one of the most common malignancies during childhood and adolescence.^[1–3]^ Meanwhile, osteosarcoma not only causes a huge disease burden on patients but also brings mental problems to their parents.^[4–9]^ Anxiety and depression are the common mental problem of parents of child cancer patients, which not only lead to poor physical health for parents of child cancer patients but also result in an unfavorable prognosis for child cancer patients.^[10–14]^ Considering that parents play a crucial role in the health care of childhood and adolescence patients with osteosarcoma in a long-term period, addressing their psychosocial issues is urgent.^[11]^

Accumulating studies have reported that the anxiety and depression of parents of child cancer patients are changing over time.^[15–17]^ For instance, the severe depression symptoms persistently exist over the 5 years among mothers of child cancer patients^[15]^; moreover, the status of anxiety and depression tend to be improved over time for parents of child cancer patients from diagnosis to 5 years^[17]^; furthermore, it also has been reported that anxiety severity is lower in father of child cancer patients compared to mother, and which is not changed across 20 months after patients’ diagnosis.^[18]^ However, the data about the longitudinal change of anxiety and depression among parents of childhood and adolescence patients with osteosarcoma is scarce.

Apart from that, exploration of risk factors of anxiety and depression is crucial to improve the management of anxiety and depression among parents of child cancer patients, and several studies have investigated related issues.^[9,19–21]^ For instance, it has been reported that economic fragility and unemployment elevate the risk of depression symptoms among parents of children patients with blood cancer^[19]^; moreover, interesting research has presented that declined income and credit rating problems are correlated with elevated risk of depression among parents of children with brain tumor.^[9]^ While the risk factors of anxiety and depression in parents of childhood and adolescence patients with osteosarcoma are unclear.

Therefore, the current study aimed to explore the longitudinal change of anxiety and depression and their related factors among parents of childhood and adolescence patients with osteosarcoma during a 3-year follow-up.

## 2. Methods

### 2.1. Participants

This study included a total of 56 childhood and adolescence patients with osteosarcoma who underwent surgery from March 2015 to April 2018, as well as 104 parents of the patients (48 patients had 2 corresponding parents; while 8 patients had one). The inclusion criteria were: patients were diagnosed as osteosarcoma; patients were younger than 20 years old; patients underwent tumor resection; parents were willing to participate in this study and complete the planned follow-up. The exclusion criteria were: parents had history of documented psychiatric disorders parents could not correctly complete the Hospital Anxiety and Depression Scale (HADS); parents had cognitive impairments that prevented them from communicating properly. The ethics were approved by Institutional Review Board of Harbin Medical University Cancer Hospital, and all parents signed informed consent forms.

### 2.2. Baseline data collection and follow-up plan

The patient’s demographics, disease characteristics, and surgery type were recorded by the case report form. The parent’s age, relation to patients, living habits, concomitant diseases, marital status, employment status, and economic status were recorded by questionnaires. The follow-up was planned at 0.5 year, 1 year, 2 years, and 3 years after patients’ discharge with a visit window period of 1 month. During the 1st, 2nd, and 3rd year, 6 parents, 11 parents and 11 parents lost to follow-up, respectively.

### 2.3. Anxiety and depression assessment

The parents’ status of anxiety and depression was evaluated at baseline (the day of patients’ hospital discharge following the surgery) and at each follow-up. The HADS was applied to evaluate the parents’ status of anxiety and depression. The HADS consisted of 2 subscales, including HADS-Anxiety (HADS-A) and HADS-Depression (HADS-D). Each subscale with 7 items scored from 0 to 21 points. In this study, HADS-A or HADS-D score >7 was the criterion for defining anxiety or depression. The severity of anxiety or depression was classified as: 0 to 7, non-anxiety/depression; 8 to 10, mild anxiety/depression; 11 to 14, moderate anxiety/depression; 15 to 21, severe anxiety/depression.^[22]^

### 2.4. Statistical analysis

A total of 28 parents lost to follow-up in this study. Data analyses were based on the Intention-to-treat principle, and the missing measurements were processed using the last observation carried forward method. Normality determination for continuous variable was performed using the Kolmogorov-Smirnov test. For the normal distributed continuous variable, it was expressed using mean and standard deviation; for the skewed distributed continuous variable, it was expressed using median and inter-quartile range; as for the categorized variable, it was expressed using number and percentage. The changes of HADS scores over time were checked using one-way analysis of variance for repeated measurements. The changes of anxiety rate and depression rate over time were determined using Chi-square test. The changes of anxiety severity and depression severity over time were evaluated using Friedman’s test. Factors related to the risk of parents’ anxiety and depression were analyzed by logistic regression, and only factors with a *P* value <.05 in the univariate logistic regression were further included in the multivariate logistic regression with enter methods. Statistical analyses were conducted by SPSS 26.0 (IBM, Armonk, New York). Figures were plotted by GraphPad Prism 7.01 (GraphPad Software Inc., San Diego, California). A 2-tailed *P* value of <.05 was regarded as significant.

## 3. Results

### 3.1. Study flow

**Figure 1.**
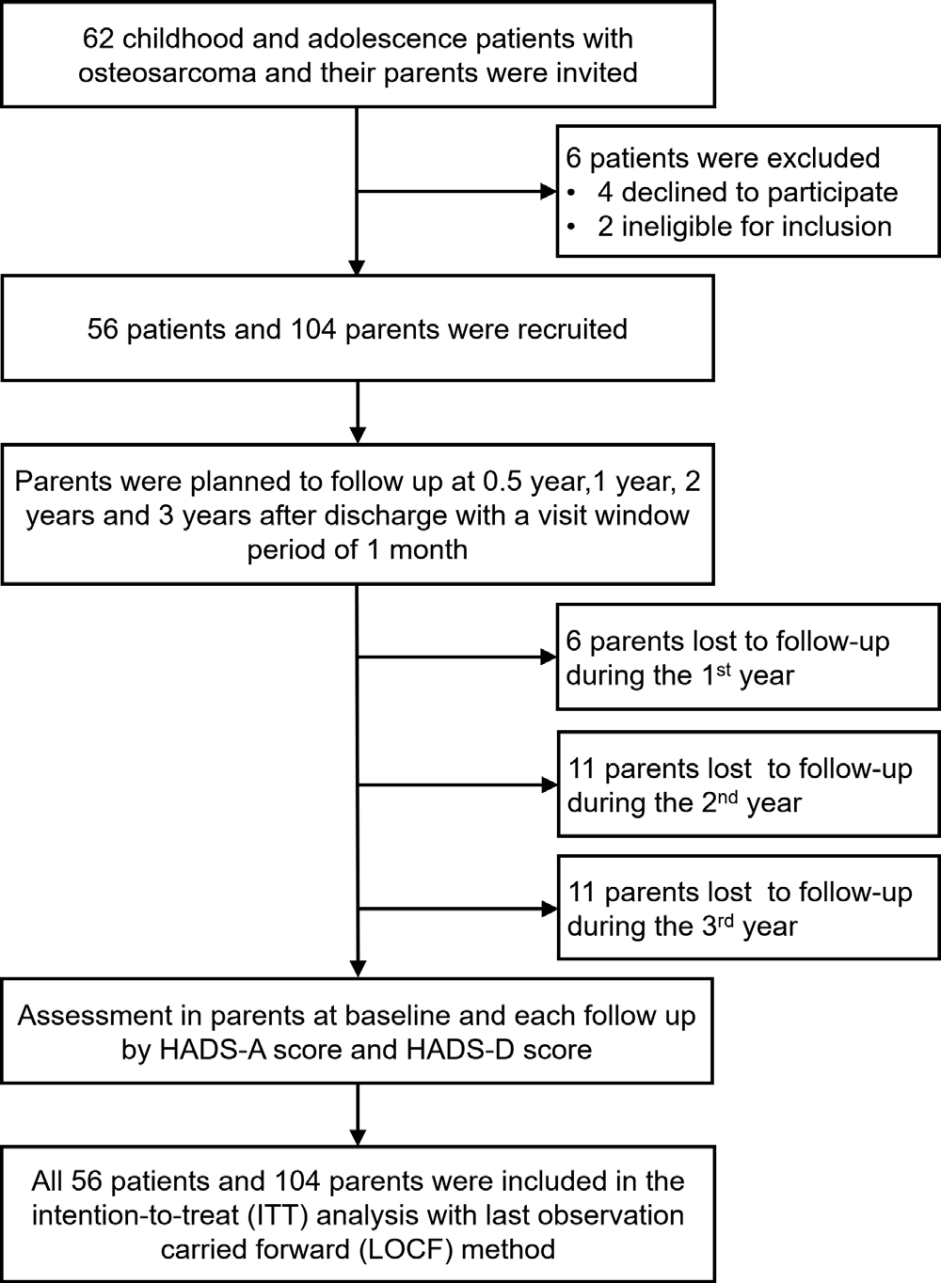
Study flow chart.

A total of 62 childhood and adolescence patients with osteosarcoma were invited, then 6 patients were excluded. Subsequently, 56 patients and 104 parents were recruited. Parents were followed up at 0.5 year, 1 year, 2 years, and 3 years after discharge with a visit window period of 1 month. During follow-up, 6, 11, and 11 parents lost to follow-up during the 1st, 2nd, and 3rd year, respectively. The parents’ status of anxiety and depression was evaluated at baseline and at each follow-up time point by HADS-A score and HADS-D score. Finally, all 56 patients and 104 parents were included in the intention-to-treat analysis with the last observation carried forward method (Fig. 1).

### 3.2. Clinical data of patients and characteristics of parents

Among 56 patients, the mean age was 12.0 ± 3.2 years; meanwhile, there were 19 (33.9%) females and 37 (66.1%) males. In terms of the Enneking stage, there were 7 (12.5%) patients with stage I; 14 (25.0%) patients with stage IIA; and 35 (62.5%) patients with stage IIB. As for surgery type, 38 (67.9%) patients received limb salvage; and 18 (32.1%) patients received amputation (Table 1).

**Table 1:** Clinical data of childhood and adolescence patients with osteosarcoma.

Items	Patients (N = 56)
Age (yrs), mean ± SD	12.0 ± 3.2
Gender, n (%)	
Female	19 (33.9)
Male	37 (66.1)
Tumor location, n (%)	
Femur	30 (53.6)
Tibia	22 (39.3)
Others	4 (7.1)
WHO classification of sarcoma, n (%)	
Conventional: chondroblastic	9 (16.0)
Conventional: osteoblastic	38 (67.9)
Conventional: other	7 (12.5)
Telangiectatic	2 (3.6)
Pathological fracture, n (%)	
No	42 (75.0)
Yes	14 (25.0)
Enneking stage, n (%)	
Stage I	7 (12.5)
Stage IIA	14 (25.0)
Stage IIB	35 (62.5)
Surgery type, n (%)	
Limb salvage	38 (67.9)
Amputation	18 (32.1)

SD = standard deviation, WHO = World Health Organization.

Among 104 parents, the mean age was 41.6 ± 5.3 years. Meanwhile, there were 55 (52.9%) mothers and 49 (47.1%) fathers. 96 (92.3%) parents were married and 8 (7.7%) parents were divorced/widowed. More detailed information about the characteristics of parents were presented in Table 2.

**Table 2:** Characteristics of parents.

Items	Parents (N = 104)
Age (yrs), mean ± SD	41.6 ± 5.3
Relation, n (%)	
Mother	55 (52.9)
Father	49 (47.1)
Smoker, n (%)	
No	42 (40.4)
Yes	62 (59.6)
Drinker, n (%)	
No	62 (59.6)
Yes	42 (40.4)
Hypertension, n (%)	
No	89 (85.6)
Yes	15 (14.4)
Hyperlipidemia, n (%)	
No	98 (94.2)
Yes	6 (5.8)
Diabetes, n (%)	
No	98 (94.2)
Yes	6 (5.8)
Marital status, n (%)	
Married	96 (92.3)
Divorced/widowed	8 (7.7)
Employment status before surgery, n (%)	
Employed	88 (84.6)
Unemployed	16 (15.4)
Level of education, n (%)	
Primary school or less	6 (5.8)
High school	45 (43.3)
Undergraduate	43 (41.3)
Graduate or above	10 (9.6)
Location, n (%)	
Urban	90 (86.5)
Rural	14 (13.5)
Family annual income (CNY), n (%)	
<10000	2 (1.9)
10000-29999	26 (25.1)
30000-49999	38 (36.5)
>50000	38 (36.5)

CNY = China Yuan, SD = standard deviation.

### 3.3. Change of anxiety at different time points among parents

**Figure 2.**
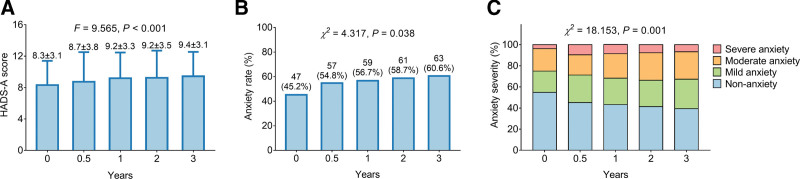
Change of anxiety. Change of HADS-A score (A), anxiety rate (B), and anxiety severity (C) from baseline to 3^rd^ year. HADS-A = HADS-anxiety.

HADS-A score presented an increasing trend with time (0 year: 8.3 ± 3.1, 0.5 year: 8.7 ± 3.8, 1 year: 9.2 ± 3.3, 2 years: 9.2 ± 3.5, 3 years: 9.4 ± 3.1) (*P* < .001) (Fig. 2A). Meanwhile, the anxiety rate was elevated with time (0 year: 45.2%, 0.5 year: 54.8%, 1 year: 56.7%, 2 years: 58.7%, 3 years: 60.6%) (*P* < .001) (*P* = .038) (Fig. 2B). Besides, anxiety severity aggravated with time (*P* = .001) (Fig. 2C).

### 3.4. Risk factors of parents’ anxiety at baseline and 3rd year

Multivariate logistic regression analysis presented that the role of mother in parents’ relation (vs father) (odds ratio (OR) = 3.160, *P* = .022), divorced or widowed marital status of parents (vs married) (OR = 12.381, *P* = .034), Enneking stage IIB (vs stage I) of patients (OR = 8.077, *P* = .018) and amputation as surgery of patients (vs limb salvage) (OR = 2.977, *P* = .042) were independently correlated with an elevated risk of parents’ baseline anxiety; however, family annual income ranging from 30000 to 49999 CNY (vs <30000 CNY) (OR = 0.189, *P* = .008) and > 50000 CNY (vs <30000 CNY) (OR = 0.120, *P* = .001) were independently correlated with declined risk of parents’ baseline anxiety (Table 3).

**Table 3:** Factors related to the risk of parents’ baseline anxiety by logistic regression model analysis.

Items	*P* value	OR	95%CI	
			Lower	Upper
**Univariate logistic regression**				
**Parents’ characteristics**				
Age	.566	1.022	0.950	1.099
Relation (Mother vs Father)	**.016**	2.664	1.197	5.931
Smoker (Yes vs No)	.418	0.722	0.329	1.588
Drinker (Yes vs No)	.694	0.853	0.388	1.878
Hypertension (Yes vs No)	.219	2.013	0.660	6.140
Hyperlipidemia (Yes vs No)	.808	1.227	0.236	6.384
Diabetes (Yes vs No)	.291	2.558	0.447	14.628
Marital status (Divorced or widowed vs Married)	**.036**	9.800	1.160	82.814
Employment status before surgery (Unemployed vs Employed)	.137	2.297	0.767	6.881
Level of education				
High school or less	Ref.			
Undergraduate	.863	1.074	0.477	2.420
Graduate or above	.130	0.281	0.054	1.456
Location (Rural vs Urban)	.338	1.744	0.559	5.439
Family annual income				
<30000 CNY	Ref.			
30000-49999 CNY	.100	0.426	0.154	1.179
>50000 CNY	**.001**	0.169	0.058	0.494
**Patients’ characteristics**				
Age	.382	1.056	0.935	1.193
Gender (Male vs Female)	.911	1.047	0.465	2.360
Tumor location				
Femur	Ref.			
Tibia	.805	0.903	0.402	2.030
Others	.858	0.865	0.177	4.228
WHO classification of sarcoma, n (%)				
Conventional: chondroblastic	Ref.			
Conventional: osteoblastic	.175	2.205	0.704	6.913
Conventional: other	.603	1.500	0.325	6.918
Telangiectatic	.999	3.9 × 10^9^	<0.001	NA
Pathological fracture (Yes vs No)	.308	1.591	0.652	3.883
Enneking stage				
Stage I	Ref.			
Stage IIA	.142	3.600	0.651	19.902
Stage IIB	**.014**	7.200	1.493	34.726
Surgery type (Amputation vs Limb salvage)	**.011**	3.029	1.284	7.143
**Multivariate logistic regression**				
Relation (Mother vs Father)	**.022**	3.160	1.177	8.484
Marital status (Divorced or widowed vs Married)	**.034**	12.381	1.208	126.863
Family annual income				
<30000 CNY	Ref.			
30000-49999 CNY	**.008**	0.189	0.055	0.652
>50000 CNY	**.001**	0.120	0.034	0.427
Enneking stage				
Stage I	Ref.			
Stage IIA	.263	3.052	0.432	21.549
Stage IIB	**.018**	8.077	1.428	45.680
Surgery type (Amputation vs Limb salvage)	**.042**	2.977	1.042	8.503

CI = confidence interval, CNY = China Yuan, NA = not available, OR = odds ratio, WHO = World Health Organization.

In addition, Enneking stage IIB (vs stage I) (OR = 5.159, *P* = .027) of patients was independently correlated with an elevated risk of parents’ 3-year anxiety; while family annual income ranging from 30000 to 49999 CNY (vs <30000 CNY) (OR = 0.303, *P* = .050) and > 50000 CNY (vs <30000 CNY) (OR = 0.255, *P* = .024) were independently correlated with declined risk of parents’ 3-year anxiety (Table 4).

**Table 4:** Factors related to the risk of parents’ 3-year anxiety by logistic regression model analysis.

Items	*P* value	OR	95%CI	
			Lower	Upper
**Univariate logistic regression**				
**Parents’ characteristics**				
Age	.941	0.997	0.926	1.074
Relation (Mother vs Father)	.062	2.146	0.964	4.778
Smoker (Yes vs No)	.065	0.455	0.198	1.049
Drinker (Yes vs No)	.820	1.098	0.492	2.452
Hypertension (Yes vs No)	.603	1.358	0.428	4.307
Hyperlipidemia (Yes vs No)	.588	0.633	0.121	3.301
Diabetes (Yes vs No)	.588	0.633	0.121	3.301
Marital status (Divorced or widowed vs Married)	.139	5.000	0.592	42.252
Employment status before surgery (Unemployed vs Employed)	.864	1.101	0.367	3.301
Level of education				
High school or less	Ref.			
Undergraduate	.532	1.307	0.565	3.024
Graduate or above	.607	0.700	0.180	2.725
Location (Rural vs Urban)	.376	1.745	0.508	5.990
Family annual income				
<30000 CNY	Ref.			
30000-49999 CNY	.125	0.418	0.137	1.272
>50000 CNY	**.013**	0.245	0.081	0.741
**Patients’ characteristics**				
Age	.108	0.900	0.792	1.023
Gender (Male vs Female)	.615	0.807	0.351	1.859
Tumor location				
Femur	Ref.			
Tibia	.565	0.784	0.343	1.794
Others	.282	0.417	0.085	2.051
WHO classification of sarcoma, n (%)				
Conventional: chondroblastic	Ref.			
Conventional: osteoblastic	.265	1.833	0.631	5.324
Conventional: other	.433	1.800	0.415	7.814
Telangiectatic	.999	1.8 × 10^9^	<0.001	NA
Pathological fracture (Yes vs No)	.563	1.314	0.521	3.315
Enneking stage				
Stage I	Ref.			
Stage IIA	.083	3.500	0.850	14.412
Stage IIB	**.010**	5.357	1.505	19.075
Surgery type (Amputation vs Limb salvage)	.197	1.782	0.741	4.289
Recurrence	**.017**	2.815	1.204	6.582
Death	**.027**	3.130	1.141	8.586
**Multivariate logistic regression**				
Family annual income				
<30000 CNY	Ref.			
30000-49999 CNY	**.050**	0.303	0.092	1.002
>50000 CNY	**.024**	0.255	0.078	0.834
Enneking stage				
Stage I	Ref.			
Stage IIA	**.070**	4.334	0.889	21.122
Stage IIB	**.027**	5.159	1.209	22.015
Patient’s recurrence	.181	2.409	0.665	8.734
Patient’s death	.874	1.132	0.244	5.240

CI = confidence interval, CNY = China Yuan, NA = not available, OR = odds ratio, WHO = World Health Organization.

### 3.5. Change of depression at different time points among parents

**Figure 3.**
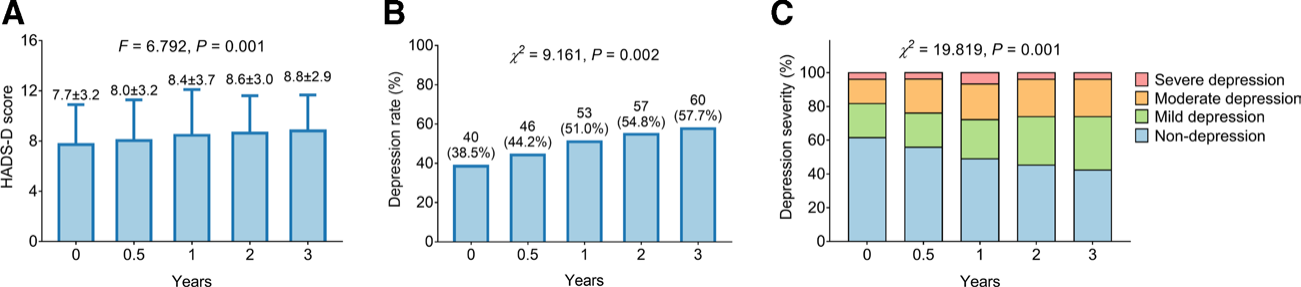
Change of depression. Change of HADS-D score (A), depression rate (B), and depression severity (C) from baseline to 3^rd^ year. HADS-D = HADS-depression.

HADS-D score presented an increasing trend from baseline to the 3^rd^ year (0 year: 7.7 ± 3.2, 0.5 year: 8.0 ± 3.2, 1 year: 8.4 ± 3.7, 2 years: 8.6 ± 3.0, 3 years: 8.8 ± 2.9) (*P* = .001) (Fig. 3A). Meanwhile, the anxiety rate was elevated from baseline to the 3^rd^ year (0 year: 38.5%, 0.5 year: 44.2%, 1 year: 51.0%, 2 years: 54.8%, 3 years: 57.7%) (*P* = .002) (Fig. 3B). Besides, anxiety severity was also increased from baseline to the 3^rd^ year (*P* = .001) (Fig. 3C).

### 3.6. Risk factors of parents’ depression at baseline and 3^rd^ year

Multivariate logistic regression analysis presented that divorced or widowed marital status of parents (vs married) (OR = 17.452, *P* = .018) and amputation as surgery of patients (vs limb salvage) (OR = 3.290, *P* = .026) were independently correlated with increased risk of parents’ baseline depression (Table 5). In addition, amputation as surgery of patients (vs limb salvage) (OR = 3.245, *P* = .022) was independently correlated with an elevated risk of parents’ 3-year depression (Table 6).

**Table 5:** Factors related to the risk of parents’ baseline depression by logistic regression model analysis.

Items	*P* value	OR	95%CI	
			Lower	Upper
**Univariate logistic regression**				
**Parents’ characteristics**				
Age	.218	1.049	0.972	1.131
Relation (Mother vs Father)	.052	2.241	0.992	5.064
Smoker (Yes vs No)	.728	0.867	0.388	1.936
Drinker (Yes vs No)	.449	1.364	0.611	3.043
Hypertension (Yes vs No)	.482	1.485	0.493	4.469
Hyperlipidemia (Yes vs No)	.165	3.444	0.601	19.749
Diabetes (Yes vs No)	.791	0.789	0.138	4.522
Marital status (Divorced or widowed vs Married)	**.017**	13.364	1.577	113.266
Employment status before surgery (Unemployed vs Employed)	.521	0.688	0.220	2.152
Level of education				
High school or less	Ref.			
Undergraduate	.053	2.292	0.988	5.314
Graduate or above	.476	0.547	0.104	2.872
Location (Rural vs Urban)	.717	1.235	0.395	3.867
Family annual income				
<30000 CNY	Ref.			
30000-49999 CNY	1.000	1.000	0.377	2.655
>50000 CNY	**.008**	0.226	0.075	0.682
**Patients’ characteristics**				
Age	.383	1.057	0.933	1.198
Gender (Male vs Female)	**.015**	3.111	1.241	7.797
Tumor location				
Femur	Ref.			
Tibia	.126	0.513	0.218	1.206
Others	.929	0.930	0.190	4.547
WHO classification of sarcoma, n (%)				
Conventional: chondroblastic	Ref.			
Conventional: osteoblastic	.064	4.333	0.918	20.465
Conventional: other	**.001**	25.000	3.522	177.477
Telangiectatic	.060	15.000	0.896	251.056
Pathological fracture (Yes vs No)	.642	0.803	0.318	2.027
Enneking stage				
Stage I	Ref.			
Stage IIA	.577	1.500	0.361	6.230
Stage IIB	.393	1.731	0.491	6.096
Surgery type (Amputation vs Limb salvage)	**.007**	3.231	1.370	7.622
**Multivariate logistic regression**				
Marital status (Divorced or widowed vs Married)	**.018**	17.452	1.632	186.590
Family annual income				
<30000 CNY	Ref.			
30000-49999 CNY	.982	0.987	0.302	3.228
>50000 CNY	.430	0.549	0.124	2.431
Patient’s gender (Male vs Female)	.062	3.262	0.941	11.304
WHO classification of sarcoma, n (%)				
Conventional: chondroblastic	Ref.			
Conventional: osteoblastic	.257	3.263	0.422	25.206
Conventional: other	.067	10.397	0.851	127.084
Telangiectatic	.343	6.172	0.144	264.771
Surgery type (Amputation vs Limb salvage)	**.026**	3.290	1.152	9.392

CI = confidence interval, CNY = China Yuan, OR = odds ratio, WHO = World Health Organization.

**Table 6:** Factors related to the risk of parents’ 3-year depression by logistic regression model analysis.

Items	*P* value	OR	95%CI	
			Lower	Upper
**Univariate logistic regression**				
**Parents’ characteristics**				
Age	.365	0.966	0.897	1.041
Relation (Mother vs Father)	.614	1.222	0.560	2.665
Smoker (Yes vs No)	.264	0.632	0.283	1.413
Drinker (Yes vs No)	.756	1.134	0.512	2.512
Hypertension (Yes vs No)	.141	0.432	0.141	1.321
Hyperlipidemia (Yes vs No)	.695	0.719	0.138	3.745
Diabetes (Yes vs No)	.999	<0.001	<0.001	NA
Marital status (Divorced or widowed vs Married)	.314	2.333	0.448	12.154
Employment status before surgery (Unemployed vs Employed)	.500	0.692	0.238	2.015
Level of education				
High school or less	Ref.			
Undergraduate	.872	1.071	0.468	2.449
Graduate or above	.280	0.467	0.117	1.860
Location (Rural vs Urban)	.964	0.974	0.312	3.041
Family annual income				
<30000 CNY	Ref.			
30000-49999 CNY	.482	0.686	0.239	1.963
>50000 CNY	**.020**	0.291	0.103	0.825
**Patients’ characteristics**				
Age	.097	0.898	0.791	1.020
Gender (Male vs Female)	.353	0.675	0.294	1.548
Tumor location				
Femur	Ref.			
Tibia	.089	0.489	0.214	1.116
Others	.241	0.385	0.078	1.900
WHO classification of sarcoma, n (%)				
Conventional: chondroblastic	Ref.			
Conventional: osteoblastic	.082	2.655	0.882	7.989
Conventional: other	.072	4.125	0.883	19.273
Telangiectatic	.999	3.0 × 10^9^	<0.001	NA
Pathological fracture (Yes vs No)	1.000	1.000	0.407	2.456
Enneking stage				
Stage I	Ref.			
Stage IIA	.359	1.867	0.492	7.085
Stage IIB	.228	2.051	0.638	6.596
Surgery type (Amputation vs Limb salvage)	**.037**	2.593	1.058	6.353
Recurrence	**.038**	2.385	1.048	5.425
Death	**.034**	2.846	1.083	7.481
**Multivariate logistic regression**				
Family annual income				
<30000 CNY	Ref.			
30000-49999 CNY	.296	0.549	0.178	1.689
>50000 CNY	.063	0.355	0.119	1.059
Surgery type (Amputation vs Limb salvage)	**.022**	3.245	1.183	8.905
Patient’s recurrence	.830	1.147	0.327	4.018
Patient’s death	.126	3.284	0.717	15.035

CI = confidence interval, CNY = China Yuan, NA = not available, OR = odds ratio, WHO = World Health Organization.

## 4. Discussion

Regarding the status of anxiety and depression among parents of children with cancer, it has been reported that anxiety and depression deteriorate among parents of children with acute lymphoblastic leukemia and central nervous system cancer^[23]^; moreover, it also has been reported that symptoms of anxiety and depression tend to be alleviated over time for parents of child cancer patients from diagnosis to 5 years.^[17]^ However, the data about the longitudinal change of anxiety and depression among parents of childhood and adolescence patients with osteosarcoma is scarce. In the current study, the status of anxiety and depression deteriorated from the baseline to the 3^rd^ year among parents of childhood and adolescence patients with osteosarcoma, which was reflected by elevated HADS-A score (from 8.3 ± 3.1 to 9.4 ± 3.1) and HADS-D score (from 7.7 ± 3.2 to 8.8 ± 2.9), increased anxiety rate (from 45.2% to 60.6%) and depression rate (from 38.5% to 57.7%), as well as elevated severity of anxiety and depression. The possible explanations might be that the parents might face the challenge of the care process for childhood and adolescence patients with osteosarcoma, including financial difficulties, patients’ feeling of social isolation by their peers, and role transition; therefore, the anxiety and depression of the parents might continue to deteriorate.^[24]^

In terms of the risk factors of anxiety and depression among parents of children with cancer, previous studies have reported that economic fragility, unemployment, parental fear of progression are correlated with elevated incidence of anxiety and depression among parents of children with blood cancer and central nervous system cancer.^[19,21]^ In the current study, factors related to the risk of anxiety and depression among parents of childhood and adolescence patients with osteosarcoma were explored, which presented that the role of the mother in parents’ relation, divorced or widowed marital status of parents, higher Enneking stage of patients, amputation as surgery of patients were independently correlated with increased risk of anxiety, while elevated family annual income was independently correlated with declined risk of anxiety. Meanwhile, divorced or widowed marital status of parents and amputation as surgery of patients were independently correlated with an elevated risk of depression. The possible explanations might be that: mother might be more emotional and vulnerable, as well as profoundly affected by the health situation of their children; thus, the role of the mother was an independent factor for the occurrence of anxiety.^[25]^ Childhood and adolescence patients with osteosarcoma who had a higher Enneking stage might suffer more from the disease and at a high risk of mortality, which increased the risk of anxiety for their parents; thus, Enneking stage IIB (vs stage I) were independently correlated with an elevated risk of parents’ anxiety.^[24]^ Parents of childhood and adolescence patients with osteosarcoma who had elevated annual income might face less financial burden on subsequent care for patients, which could alleviate parents’ anxiety^[9]^; thus, family annual income ranging from 30000 to 49999 CNY and > 50000 CNY were independently correlated with the declined risk of parents’ anxiety. Divorced or widowed marital status of parents might face more burdens such as financial difficulties and caregiving strain for childhood and adolescence patients with osteosarcoma, which could result in more likelihood of anxiety and depression among parents.^[9]^ Childhood and adolescence patients with osteosarcoma who receive amputation as surgery might suffer more post-surgical physical problems and psychological issues, which could worsen anxiety and depression among their parents; thus, amputation as surgery was independently correlated with increased risk for anxiety and depression.^[26]^

There existed several limitations in the current study: the sample size was relatively small, which could be enlarged in further study; we only included the patients who underwent tumor resection, thus, patients receiving other treatments such as chemotherapy and radiotherapy could be enrolled in the future; this was a single-centered study, thus the enrolled parents came from the similar area, which might result in bias in discovery.

To be conclusive, anxiety and depression deteriorate with time among parents of childhood and adolescence patients with osteosarcoma, which are affected by parental role, marital status, family annual income, surgery type, and Enneking stage.

## Author contributions

**Conceptualization:** Honghe Li.

**Data curation:** Jie Meng.

**Formal analysis:** Jie Meng, Jing Wu, Xinying Zhang, Libo Guo.

**Investigation:** Jie Meng, Jing Wu, Libo Guo.

**Methodology:** Jie Meng, Jing Wu, Xinying Zhang.

**Resources:** Jing Wu, Xinying Zhang, Libo Guo.

**Supervision:** Honghe Li.

**Validation:** Honghe Li.

**Writing – original draft:** Xinying Zhang, Libo Guo.

**Writing – review & editing:** Libo Guo, Honghe Li.
